# Spatial Configuration of Drought Disturbance and Forest Gap Creation across Environmental Gradients

**DOI:** 10.1371/journal.pone.0157154

**Published:** 2016-06-08

**Authors:** Margaret E. Andrew, Katinka X. Ruthrof, George Matusick, Giles E. St. J. Hardy

**Affiliations:** 1 Environmental and Conservation Sciences, School of Veterinary and Life Sciences, Murdoch University, Murdoch, Western Australia 6150, Australia; 2 Centre of Excellence for Climate Change, Woodland and Forest Health, Murdoch University, Murdoch, Western Australia 6150, Australia; Chinese Academy of Forestry, CHINA

## Abstract

Climate change is increasing the risk of drought to forested ecosystems. Although drought impacts are often anecdotally noted to occur in discrete patches of high canopy mortality, the landscape effects of drought disturbances have received virtually no study. This study characterized the landscape configuration of drought impact patches and investigated the relationships between patch characteristics, as indicators of drought impact intensity, and environmental gradients related to water availability to determine factors influencing drought vulnerability. Drought impact patches were delineated from aerial surveys following an extreme drought in 2011 in southwestern Australia, which led to patchy canopy dieback of the Northern Jarrah Forest, a Mediterranean forest ecosystem. On average, forest gaps produced by drought-induced dieback were moderate in size (6.6 ± 9.7 ha, max = 85.7 ha), compact in shape, and relatively isolated from each other at the scale of several kilometers. However, there was considerable spatial variation in the size, shape, and clustering of forest gaps. Drought impact patches were larger and more densely clustered in xeric areas, with significant relationships observed with topographic wetness index, meteorological variables, and stand height. Drought impact patch clustering was more strongly associated with the environmental factors assessed (R^2^ = 0.32) than was patch size (R^2^ = 0.21); variation in patch shape remained largely unexplained (R^2^ = 0.02). There is evidence that the xeric areas with more intense drought impacts are ‘chronic disturbance patches’ susceptible to recurrent drought disturbance. The spatial configuration of drought disturbances is likely to influence ecological processes including forest recovery and interacting disturbances such as fire. Regime shifts to an alternate, non-forested ecosystem may occur preferentially in areas with large or clustered drought impact patches. Improved understanding of drought impacts and their patterning in space and time will expand our knowledge of forest ecosystems and landscape processes, informing management of these dynamic systems in an uncertain future.

## Introduction

Ecological disturbances are known to be important drivers of the patch-mosaic structure of vegetation communities across a landscape. Because disturbances occur heterogeneously in space and time, they produce variability in landscape composition, influencing patterns of the types of vegetation present, as well as their successional stages and vertical structure, among other factors [1]. In addition, the spatial configuration of landscapes is strongly dependent on disturbances, and varies between disturbance agents and disturbance regimes. For example, the size, shape [2], and spatial patterning [3] of forest gaps depends on whether they were produced by wind, fire, or biological agents. Further, for a given disturbance agent, variation in disturbance regime due to differences in environment [4] or management (such as fire suppression [5–7]) produces differences in the spatial characteristics of disturbed patches.

One type of disturbance that has received surprisingly little consideration as a driver of landscape structure is drought. Drought-induced mortality has been implicated in several studies of forest gap creation [8–10], but much less frequently than other gap formation agents such as windthrow, pests, or pathogens (e.g., only one of the papers reviewed in [11] identified drought as the cause of gap formation). Yet drought is gaining widespread research attention as a formidable cause of forest mortality, one that is likely to increase more in prevalence, severity, and importance under climate change [12–15]. Many drought-induced dieback and tree mortality events have already been observed worldwide, in diverse forest systems [16–17].

Indeed, drought clearly does have important impacts on forest landscape structure. Many of the drought-induced forest disturbances reported to date are described as having been extremely patchy in space, characterized by discrete areas of high mortality interspersed throughout a larger forested landscape: patchy drought-induced tree mortality and dieback events have been observed in systems as spatially and environmentally dispersed as semi-arid woodlands of the southwest United States [18–19] and South Africa [20], Australian savannas [21–22], Mediterranean-type forest ecosystems in Spain [23] and southwestern Australia [24–25], temperate *Nothofagus* forests in Patagonia [26], and aspen stands in the Canadian boreal forest [27–28] and Colorado [29]. Yet, although the patchy configuration of drought impacts is often anecdotally noted, drought impact patches are rarely characterized. Instead, studies of drought-induced mortality have almost exclusively focused on drought in the context of *landscape composition*. In other words, they have investigated where drought-induced mortality occurs in a landscape and why, modeling the presence/absence of drought impact patches or percentage of tree mortality within a plot as a function of the physical and biological characteristics of those sites, with varying conclusions. In some landscapes, drought impacts have occurred on the xeric portions of water availability gradients associated with climate [28–31], soil properties [18, 22], and geological substrates [23]. Conversely, elsewhere, drought disturbance has occurred in locations that have historically experienced relatively greater water availability than their surroundings [32], possibly because individuals there were not acclimated to drought [33] or because the stand characteristics that had developed in those areas were not sustainable under severe water stress [34–35].

However, the *landscape configuration* aspects of drought impacts and their associations with environmental gradients remain largely unknown. But there are many reasons to be interested in the effects of drought on landscape configuration as well as on composition. Patch and landscape structure due to disturbances can have important effects on the species inhabiting forests [36–41]. Thus, the spatial characteristics of drought impact patches may influence biodiversity beyond the direct effects of the mortality event. In addition, patch structure has been suggested to indicate an impending ecological regime shift to an alternative stable state [42–45]. Consequently, the spatial patterning of current drought-induced mortality events may suggest the proximity of ecological tipping points to a non-forested state, a possible outcome of increasing drought stress under climate change for which there is growing concern [16, 46–47].

Finally, we suggest that the spatial properties of drought impact patches may provide quantitative measures of the intensity of drought impacts that offer greater nuance and, potentially, greater ecological understanding than simply the presence/absence of drought-induced mortality. Portions of the landscape that are more vulnerable to drought are generally expected to experience more extensive damage due to droughts. However, this greater extent of drought impacts may be manifested spatially in various ways: In vulnerable areas there may be a greater number of drought impact patches (or, equivalently, a higher patch density, shorter nearest neighbor distances between patches, and greater patch clustering), drought impact patches may be larger, or both. If the incidence of drought impact patches is increased in vulnerable areas, investigations into drought impact patch *presence* should successfully detect environmental associations, but observed patterns may be weakened by the occurrence of small or isolated drought impact patches elsewhere in the landscape. In contrast, spatial pattern metrics such as patch size or clustering will indicate that such impacts are minor relative to those in areas with larger or denser aggregations of drought impact patches, allowing the environmental associations of drought impacts to be more confidently identified.

The objectives of this study were to (1) characterize the spatial configuration of forest patches that experienced sudden dieback following severe drought stress, and (2) use drought impact patch characteristics as indicators of drought impact intensity in order to gain greater insights into the factors influencing drought vulnerability, and, specifically, to determine whether xeric or mesic landscape positions are more prone to drought impacts. To do so, we investigated a drought-induced forest dieback event characterized by extremely patchy canopy mortality in the Northern Jarrah Forest of southwestern Australia [25].

## Materials and Methods

### Northern Jarrah Forest drought

**Fig 1:**
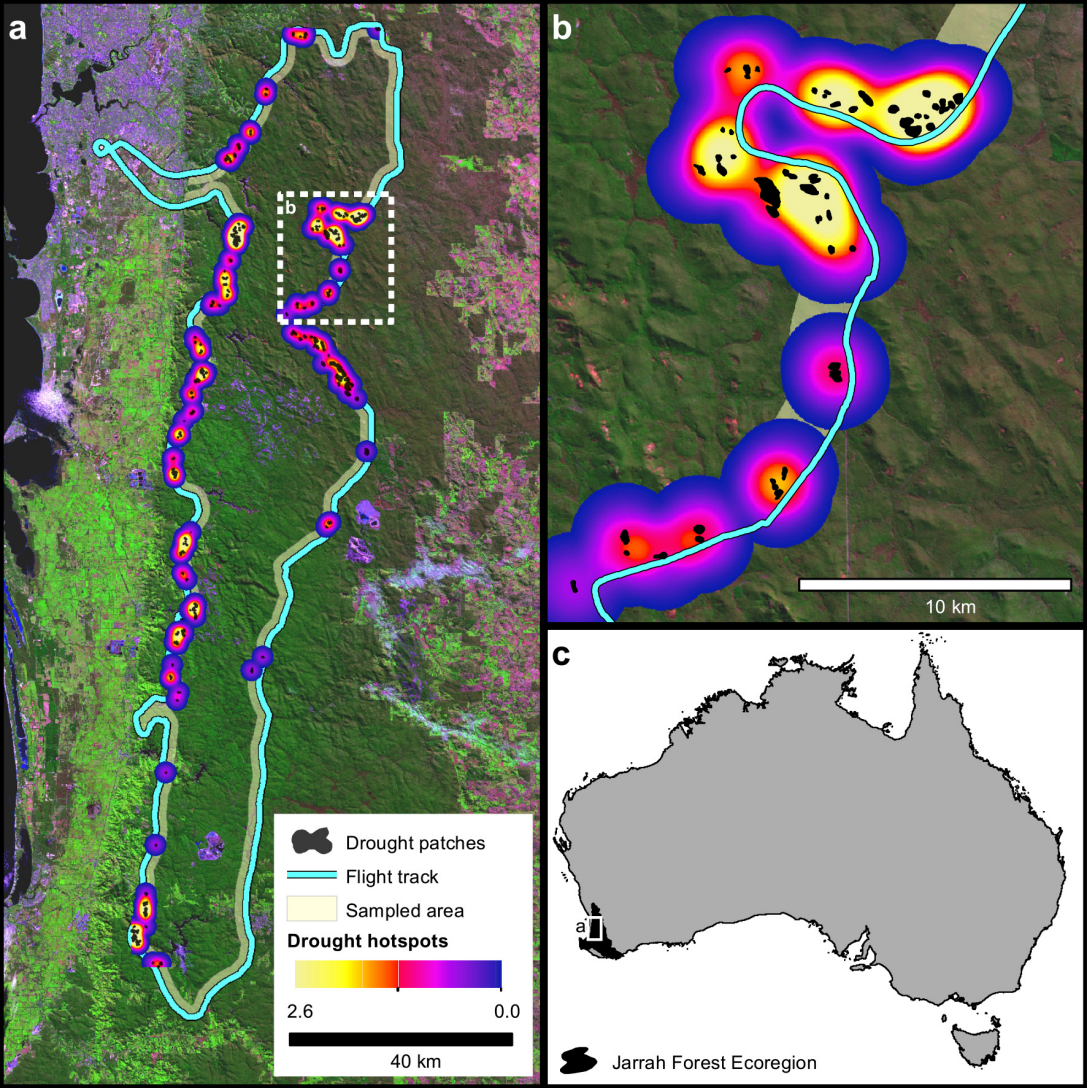
*(a)* Map of the study area, sampled flightline, and drought impact patches and patch hotspots identified in an aerial survey following the 2010/11 severe drought and canopy dieback in the Northern Jarrah Forest, with *(b)* a zoom inset providing greater detail of the variation in patch structure. *(c)* Locator map illustrating the study extent and the location of the Northern Jarrah Forest in southwestern Australia. The background in panels *(a)* and *(b)* is a near-natural color composite image of Landsat data (Landsat data available from the U.S. Geological Survey).

The Northern Jarrah Forest (NJF) spans over 1 million ha in the hills east of Perth, Western Australia (Fig 1C). Overstory species composition is dominated by jarrah (*Eucalyptus marginata*) and co-dominated by marri (*Corymbia calophylla*) trees. A part of the southwestern Australian global biodiversity hotspot, the NJF hosts great diversity of woody species in the under- and mid-stories. Unlike the surrounding regions, which have been largely converted to anthropogenic land uses, the native forest cover of the NJF has been largely maintained, although it has been extensively managed for timber and mining [48]. Over the past 150 years, virtually all of the NJF has experienced forest harvest activities at least once under diverse silvicultural practices; more recently, areas have been set aside for conservation or because of low site productivity [49]. As well, prominent natural disturbances in the NJF include fire [50], dieback due to *Phytophthora cinnamomi* [51], and climate extremes [25, 52].

Southwestern Australia (SWA) has a semi-arid Mediterranean type climate, and is considered to be among the Australian systems most vulnerable to ecological regime shifts because, in addition to other stresses, it is near the climatic tolerance limit of native ecosystems [53]. This region has already experienced climate warming—with annual average temperatures increasing by 0.15°C/decade, and drying—most notably a persistent 15–20% step-change reduction in annual rainfall since the mid-1970s [54], with consequent reductions in groundwater and streamflow [55–57]. Rainfall in SWA is associated with the El Niño—Southern Oscillation, but the nature of this association has altered over the same time frame as the observed drying pattern [58]. The current pattern of increased warming, drying, and drought stress is expected to continue with climate change [54, 57].

On top of this general pattern of increasing drought stress are further climatic disturbances, such as an extreme drought in SWA over 2010–2011: 2010 was the driest year over the instrument record, 1900-present [59], receiving 40–50% below-average rainfall, as well as the 2^nd^ warmest year on record [25]. The combined moisture stress of extreme drought and an extended heatwave in February (late summer) 2011 triggered widespread canopy dieback of the NJF [25]. Canopy dieback was spatially heterogeneous, occurring in discrete patches of complete canopy loss. Overall, more than 16,000 ha distributed over the NJF were impacted by the drought [25].

### Characterizing drought impact patches

Following the dieback event, an aerial survey of the NJF was conducted in May 2011 (autumn) to locate drought impact patches. The survey was conducted along two predominantly north-south transects (Fig 1A). Transects were selected to span north-south and east-west gradients in temperature and aridity, respectively. Although this survey design did not allow a random sampling of drought impact patches, previous work has shown that it identifies the same environmental associations of drought impacts as does a more comprehensive regional inventory and provides a generally unbiased sample of the environmental conditions of the NJF [60].

Drought impacts are distinct from other disturbance processes in terms of the timing, size, and severity of the impact, as well as the speed of the canopy’s recovery. The timing of the aerial surveys, shortly after the drought-induced dieback event, enabled distinguishing between drought impacts and canopy disturbances caused by other processes such as frost damage and dieback due to infection by *Phytophthora cinnamomi*, which occur after cold nights and during warm, wet periods, respectively, rather than hot, dry conditions. In addition, drought impact patches were visibly distinct from gaps produced by other disturbance agents, and were identifiable from uniformly brown, dry foliage in the canopy surrounded by a radiating pattern of chlorotic overstory vegetation [25]. Understory vegetation experienced even more extensive damage [25], resulting in disturbed patches that were heavily damaged/discoloured. In contrast, gaps formed by fires tend to be larger and exhibit complete charring on stems and branches. Gaps produced by insect outbreaks are often smaller and contain a more heterogeneous mixture of green, brown, and missing foliage. As well, trees tend to resprout immediately following fire, frost, and insect disturbance, while recovery following the drought was not yet evident at the time of the aerial surveys. Finally, gaps formed by tree aging generally occur at the scale of individual trees or small groups of trees rather than in more extensive patches.

Oblique aerial photos were taken of patches with drought-induced canopy damage larger than 0.3 ha within approximately 2 km of the flightline (n = 226). Locations of each photo were recorded in flight with a handheld GPS unit (GPS 92, Garmin International Inc., Missouri, USA) and photos were georeferenced to high-resolution orthophotos using readily identifiable landmarks. The boundaries of all drought impact patches were delineated from the aerial photos (Fig 1B; and see [25] for further details). Each patch was characterized by (1) its area, (2) its shape complexity, and (3) the degree of clustering of drought impact patches within its neighbourhood. All patch metrics were calculated in ArcMap (ESRI, Redlands, CA, USA), as described below.

Patch size distributions are an informative descriptor of disturbance regimes [61–62]. However, because they tend to follow highly skewed distributions with many small and few large patches, they are not well described by typical statistical summaries such as the mean and standard deviation [63]. Instead, we characterized the size distribution of drought impact patches in the NJF with the exponent of the power law distribution. A power law distribution was fit to the observed patch sizes by maximum likelihood using R code accompanying [63].

Patch shape complexity was estimated using the shape index of [64]. This metric is the ratio of the observed patch perimeter to the perimeter of a maximally compact patch of the same area. Unlike other measures of patch shape (such as perimeter or the perimeter to area ratio), it is not confounded by effects of patch size. Given the vector delineation of patch boundaries, the corresponding maximally compact patch was defined to be a circle of the same area as the observed patch.

A kernel density interpolation was used to estimate the degree of patch clustering throughout the study area (Fig 1). A 2-km kernel was used, corresponding to the width of the sampled transect, and patch densities were interpolated to a 30 m raster grid. Each patch was attributed with the density value estimated at the location of its centroid.

### Environmental associations of drought impact patch characteristics

The spatial structure of drought impacts was explored further by investigating relationships between patch characteristics (size, shape and clustering) and environmental gradients influencing water availability. Three sets of environmental variables derived from readily available spatial datasets were considered, related to (1) meteorological/hydrological drivers of water availability, (2) topographic drivers of water availability, and (3) stand characteristics influencing water use (Table 1). For each variable, patches were attributed with the pixel value occurring at the location of the patch centroid.

**Table 1:** Environmental variables used to explore relationships between drought impact patch characteristics (as a measure of drought impact intensity) and moisture gradients associated with meteorology, water use, and terrain, as well as stand characteristics influencing water balance.

Variable	Scale	Source
**Meteorological / water use variables**		
Precipitation (m)	5 km	Australian Water Availability Project water balance model inputs [65]
Average minimum temperature (°C)	5 km	Australian Water Availability Project water balance model inputs [65]
Average maximum temperature (°C)	5 km	Australian Water Availability Project water balance model inputs [65]
Potential evapotranspiration (m)	5 km	Australian Water Availability Project water balance model outputs [66]
Actual evapotranspiration (m)	5 km	Australian Water Availability Project water balance model outputs [66]
Actual evapotranspiration / Potential evapotranspiration	5 km	Australian Water Availability Project water balance model outputs [66]
Potential evapotranspiration—Actual evapotranspiration (m)	5 km	Australian Water Availability Project water balance model outputs [66]
**Topographic variables**		
Elevation (m)	30 m	Shuttle radar topography mission [67, 68]
Topographic wetness index	90 m	Shuttle radar topography mission [69]
**Stand variables**		
Tree cover	250 m	MODIS Vegetation Continuous Fields % tree cover [70]
Stand height	1 km	Spaceborne lidar + auxiliary environmental variables [71]

Interpolated surfaces of meteorological and hydrological variables were provided by the Australian Water Availability Project (AWAP [65–66]) at monthly resolution over the years 1975–2011. From this dataset, variables were selected to span weather conditions (precipitation, average daily temperature minima and maxima, and potential evapotranspiration) and water use (actual evapotranspiration and transpiration), as well as composite variables relating actual to potential evapotranspiration. However, the appropriate temporal resolution over which to estimate the water availability variables was unknown. In other words, was the drought-induced dieback the result of acute stress immediately prior to crown mortality, or rather a function of extended drought stress over longer time periods? Likewise, it was unclear if the occurrence of drought impact patches was related to generally xeric positions on the landscape (i.e., dry conditions based on climatological averages) or to the specific spatial patterning of meteorological water availability at the time of the drought. Consequently, 20 possible parameterizations of each variable spanning all factorial combinations of four temporal grains and five temporal extents (Table 2) were estimated. Of the five extents, one represented the climatological average conditions; the remaining four estimated the current conditions, either absolutely, or relative to the historic pattern. These 20 parameterizations were evaluated for their ability to explain patch characteristics with univariate (or bivariate, in the case of the ‘Current + historic as covariate’ temporal extent) regression models. For each of the seven meteorological and hydrological variables, the temporal parameterization that minimized the Akaike’s Information Criterion (AIC) of generalized linear models with a given patch characteristic was chosen for use in subsequent analyses.

**Table 2:** Temporal resolutions (grain and extent) evaluated for the meteorological/hydrological variables. All pairwise combinations of grain * extent were tested for modelling spatial variation in the characteristics of drought impact patches.

Grain	Extent
Month (FEB)	Historic averages (1975–2011)
Summer (DEC to FEB)	Current water year (2010/11)
Spring and summer (SEP to FEB)	Current as % of historic
Water year (MAR to FEB)	Current—historic anomaly
	Current + historic as covariate

Two topographic variables were used (Table 1): elevation [68] and the Topographic Wetness Index (TWI [69]), both derived from the Shuttle Radar Topography Mission (SRTM) digital elevation model [67]. The TWI estimates the expected water availability based on catchment processes, by which soil depth and soil moisture are expected to be greater in flat, downslope areas.

Finally, two stand characteristics were derived from global Earth observation data products (Table 1): percent tree cover [70] and stand height [71]. Tree cover and height may be related to general gradients of water availability, but may also be indicators of water demand at the time of the drought. While denser, taller stands may develop at generally more mesic sites, dense stands will have greater water use [35, 57, 72] and, thus, potentially greater competition for moisture; taller trees may have greater hydraulic vulnerability under extreme drought [73].

For each patch metric, generalized linear models were used to explore spatial associations with the 11 environmental variables (the optimal temporal parameterization of each meteorological/water use variable, plus the topographic and stand structure variables; Table 1). Variables were entered stepwise, with stopping criteria based on AIC. Because the addition of a single variable may slightly reduce AIC without increasing model likelihood, variable entry was terminated when no reduction in AIC greater than ΔAIC = 2.0 was possible, rather than when the minimum AIC was reached. All patch characteristics were log transformed to achieve homoscedasticity and improve normality of the residuals. Distance from the patch centroid to the flightline was also included as a possible covariate to control for systematic distortions in the oblique imagery with distance from the observer. Final models were screened to ensure that the entered variables were not highly intercorrelated. Absence of residual spatial patterns was confirmed by evaluating correlations between the model residuals and x- and y-coordinates, and with Mantel tests. All statistical analyses were conducted in R (http://cran.r-project.org).

## Results

**Fig 2:**
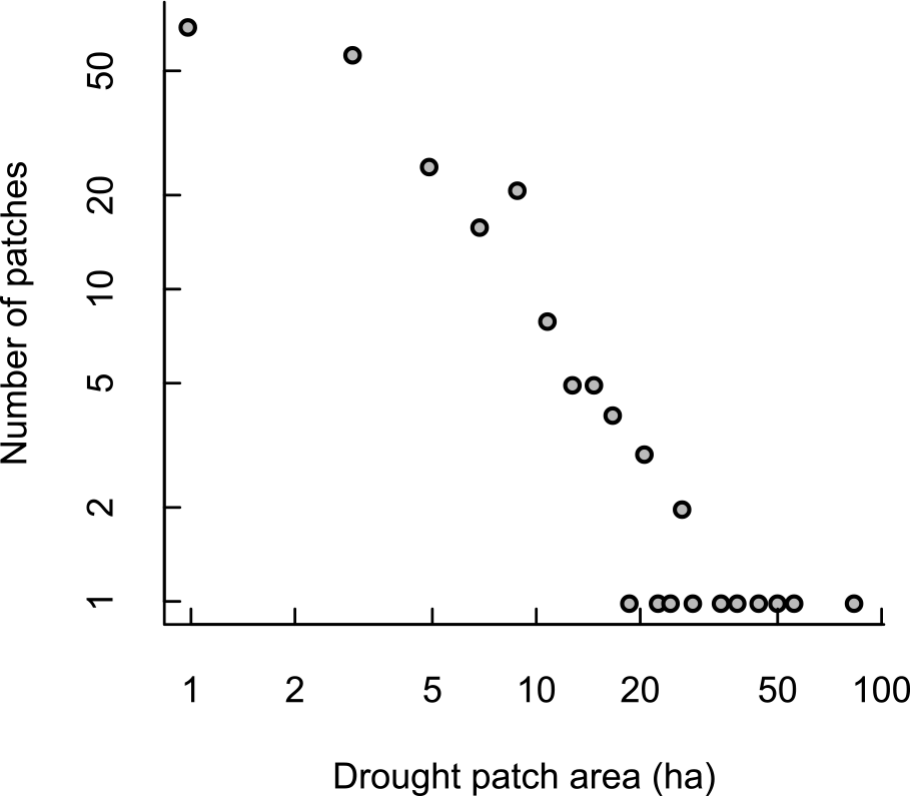
Histogram (plotted in log-log scale) of drought impact patch sizes.

There was substantial variation in the size, shape, and clustering of drought impact patches throughout the sampled area (Fig 1). Patch sizes averaged 6.6±9.7 ha (range = [0.3 ha, 85.7 ha]). The distribution of patch sizes was well fit by a power law distribution with exponent α = 2.69 (Kolmogorov-Smirnov D = 0.05, p > 0.1; Fig 2). In contrast, the observed patch size distribution differed significantly from a normal distribution (Shapiro-Wilk, p < 0.0001). Patch shapes ranged from circular (shape = 1.0) to quite irregular (shape = 2.3), but were generally fairly compact (mean±s.d. = 1.2±0.2). The kernel density estimates of drought impact patch clustering provide a relative measure of whether a patch occurred within a hotspot of drought impacts. Values are presented here to give an indication of the range of variation observed but should be interpreted relatively. On average, patches tended to be fairly isolated at 2 km scale (mean±s.d. = 0.9±0.5, min = 0.2). Kernel density peaked at 2.6 in the densest cluster of drought impact patches (the last cluster of drought impact patches in Fig 1 when traveling clockwise around the flightline before returning to the airport).

**Fig 3:**
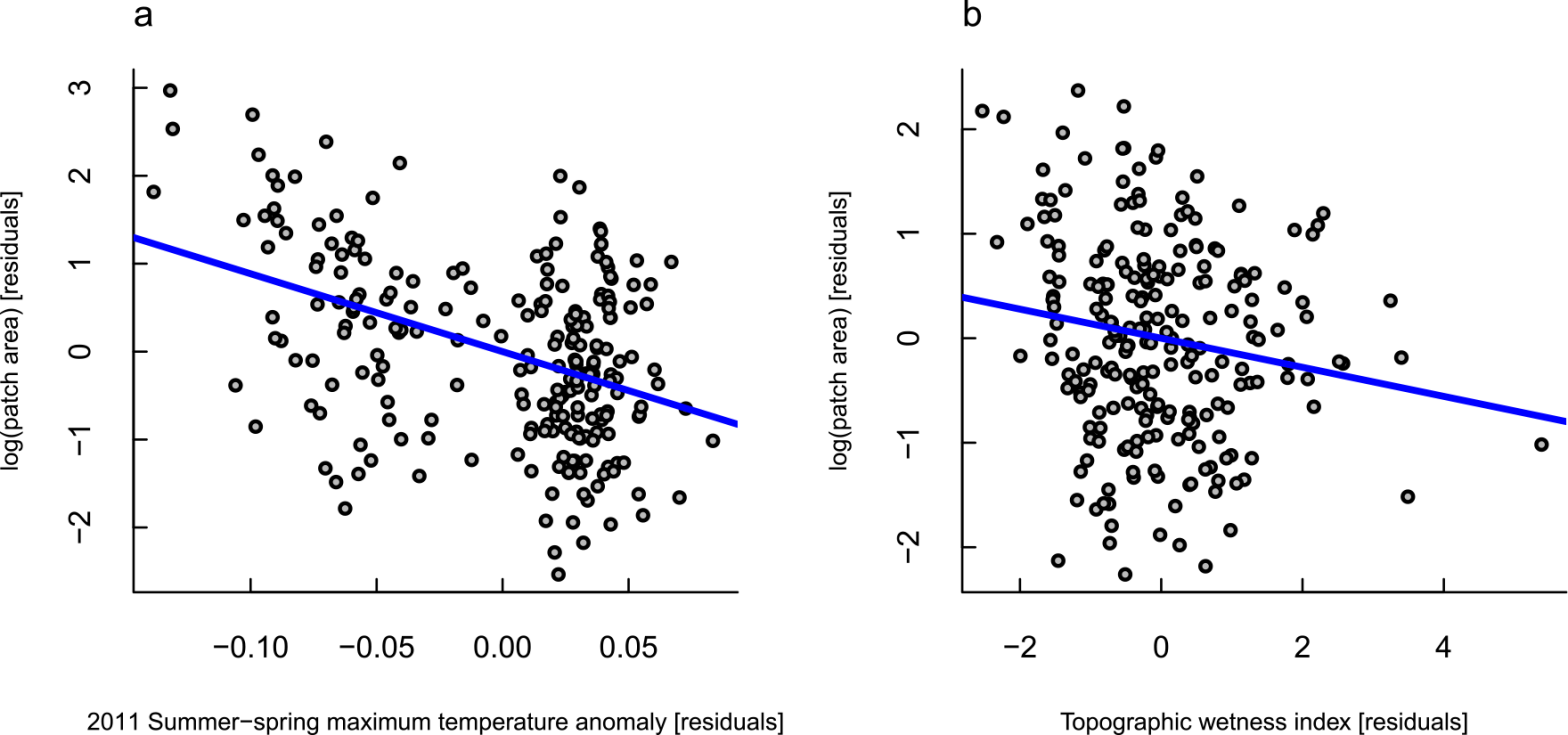
Partial-regression plots from the final regression model of drought impact patch size as a function of environmental variables related to water availability: *(a)* the independent effect of the 6-month average daily temperature maxima at the time of the drought, expressed as an anomaly from the long-term mean; and *(b)* the independent effect of the topographic wetness index on patch sizes.

**Fig 4:**
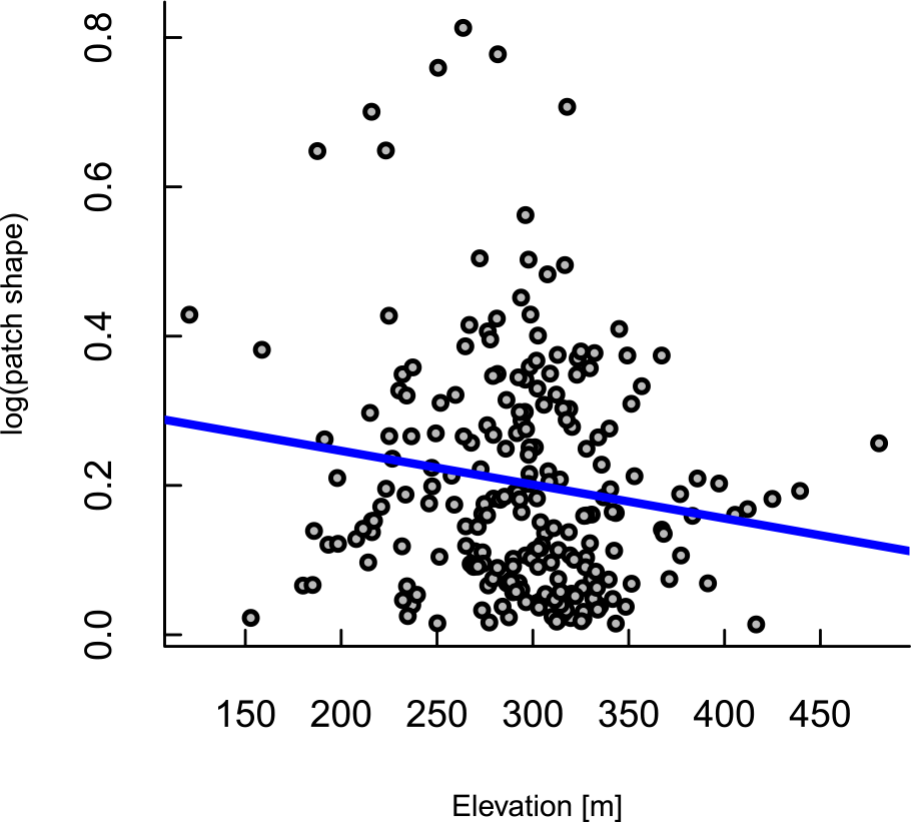
Scatterplot of the final regression model of drought impact patch shape as a function of elevation. No other environmental variables assessed were related to patch shape.

Spatial variation in patch size was associated primarily with average daily temperature maxima over the 6-months prior to the dieback, expressed as an anomaly from the historic conditions, and moderately with the topographic wetness index (Table 3). Larger patches occurred at sites that had a smaller temperature anomaly relative to the historic average and that had lower expected water availability, based on topography (Fig 3). After controlling for distortions due to distance from the flightline, 18% of variation in patch size could be explained by these two variables. Patch shape was largely unrelated to the environmental variables examined, but there was a slight tendency for more convoluted patches at lower elevations (Table 4, Fig 4).

**Table 3:** Final regression model of drought impact patch size as a function of environmental variables related to water availability. Distance of the patch to the flightline is included as a covariate to account for spatial distortions in the oblique aerial photography.

term	coefficient	SE	t	p	
intercept	27.372	2.479	11.043	< 0.0001	***
2011 summer-spring maximum temperature anomaly	-8.860	1.284	-6.901	< 0.0001	***
Topographic wetness index	-0.139	0.057	-2.456	0.01	*
Distance to flightline	0.001	0.000	3.920	0.0001	***
**R**^**2**^	**0.208**				

**Table 4:** Final regression model of drought impact patch shape as a function of elevation.

term	coefficient	SE	t	p	
intercept	0.337	0.060	5.593	< 0.0001	***
Elevation	-4.5*10^−4^	2*10^−4^	-2.253	0.03	*
**R**^**2**^	**0.022**				

**Fig 5:**
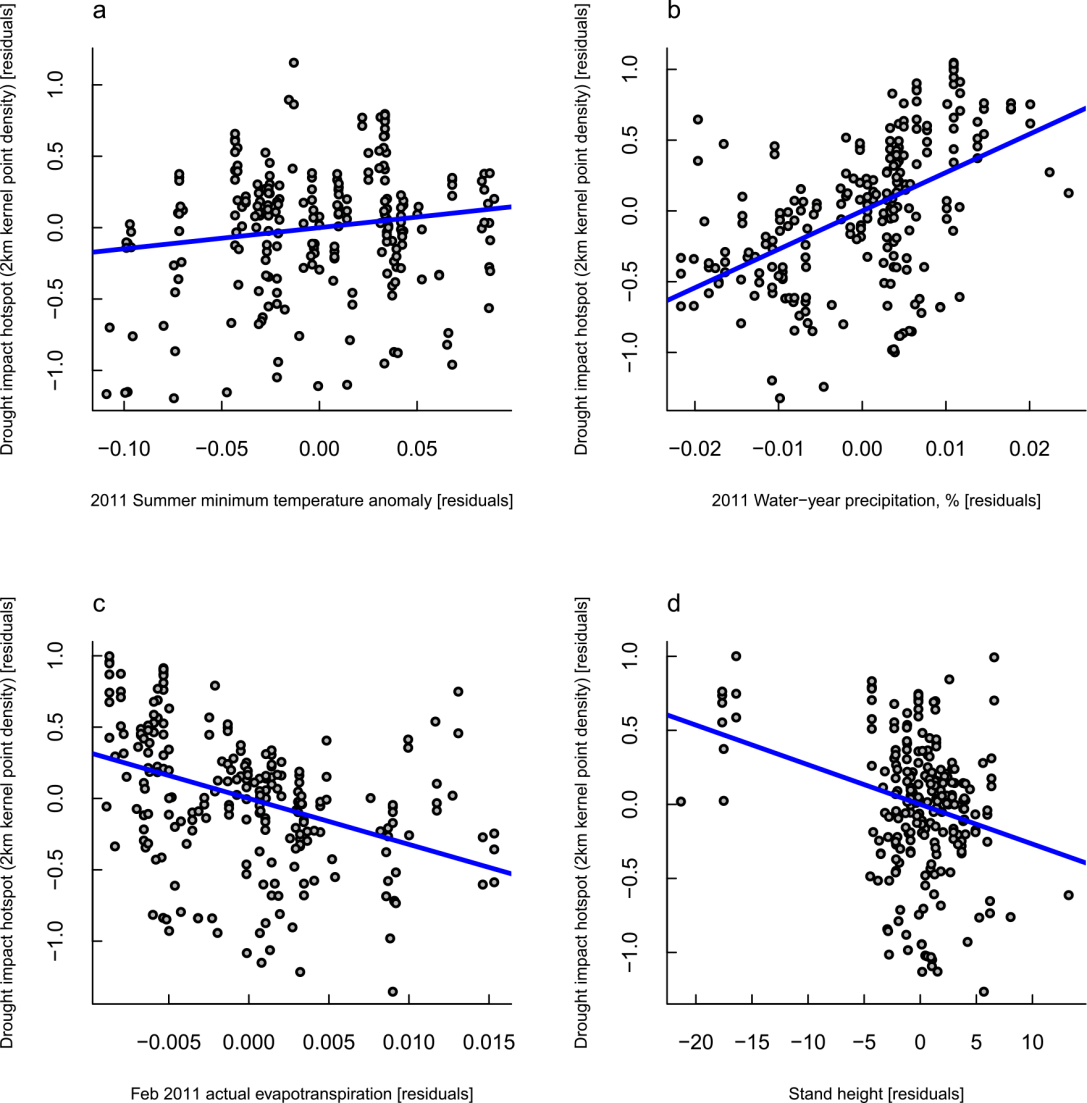
Partial-regression plots from the final regression model of drought impact patch clustering as a function of environmental variables related to water availability: Each panel plots the independent effects of *(a)* summertime average daily temperature minima at the time of the drought, expressed as an anomaly from the long-term mean; *(b)* annual precipitation in the drought year, expressed as a percentage of the long-term mean; *(c)* actual evapotranspiration in the month of the canopy dieback event; and *(d)* stand height.

The strongest environmental associations were observed for patch clustering (Table 5). Patches were parts of denser clusters at sites where precipitation in the year of the drought was more similar to the historic average, actual evapotranspiration in the month of the dieback was lowest, tree heights were shorter, and there was a greater divergence of daily temperature minima in the season of the drought from long-term averages (Fig 5). Overall, 32% of the variation in drought impact patch clustering was explained (Table 5).

**Table 5:** Final regression model of drought impact patch hotspots (2-km kernel density) as a function of environmental variables related to water availability and forest height.

term	coefficient	SE	t	p	
intercept	-16.982	1.748	-9.715	< 0.0001	***
2011 summer minimum temperature anomaly	1.482	0.642	2.310	0.02	*
2011 water-year precipitation, %	27.143	3.096	8.768	< 0.0001	***
Feb 2011 actual evapotranspiration	-32.197	5.113	-6.297	< 0.0001	***
Stand height	-0.027	0.006	-4.180	< 0.0001	***
**R**^**2**^	**0.317**				

## Discussion

### Drought gaps and landscape structure

Drought disturbance is an important gap formation agent and driver of landscape structure in forest ecosystems, as highlighted here for the Northern Jarrah Forest of southwestern Australia. Curiously, although conspicuous spatial patterning of forest drought disturbance is widely noted, it has not yet been the subject of landscape ecology research. Here we demonstrate that severe drought produces considerable variation in the patch structure of a forested landscape. The exponent of the power law distribution of patch sizes provides a useful summary of a disturbance regime [62, 74]. However, patch characteristics can vary between disturbance agents, and it can be challenging to fit a single distribution and derive inferences from forest patches that are a legacy of mixed disturbances [61, 75]. An advantage of our study is that it characterizes landscape effects from a single disturbance agent and a single disturbance event, thereby allowing the specific impacts of drought disturbance to be determined. Dieback patches resulting from the 2010/11 severe drought in the NJF followed a power law size distribution with exponent α = 2.69. This is within the range of values reported elsewhere (see reviews in [62, 74]) and is consistent with a disturbance regime dominated by small disturbance patches (i.e., α > 2).

While the concept of small gap-phase disturbance processes is a major focus of forest ecology, it has rarely been applied to semi-arid, and especially Australian forest systems, or systems traditionally considered to be dominated by large disturbances such as fire. However, forests experience multiple types of disturbances operating on different spatial and temporal scales and the characteristics of fire-driven systems do not preclude the occurrence or importance of fine-scale disturbance processes. The historical fire regime in the NJF is one of frequent, low intensity fires causing little mortality of mature trees [76–77]. Stand age structure is thus variable and mortality relatively idiosyncratic, although climatic disturbances such as extreme drought may open larger canopy gaps due to mortality of multiple trees simultaneously. In addition, the presence and spatial patterning of drought impacts may have important effects on the intensity and propagation of fire across a landscape [78]. Another factor that is likely related to the limited historic consideration of gap dynamics in these systems is extensive recovery by vegetative regeneration [79]. However, resprouting ability is not restricted to fire adapted systems [80] and is a major contributor to forest recovery even in the temperate forests of classical forest gap theory [81]. Gap dynamics, such as caused by drought-induced dieback, are likely to be important in all forest systems, regardless of the dominant (by area) disturbance agent.

### Environmental associations of patch characteristics and gap dynamics

There are two broad mechanisms that may lead to drought impacts, each giving distinct expectations of which sites in a landscape will be most vulnerable to drought, and previous studies have found support for both of them (see Introduction). In the first, drought-induced dieback is a function of abiotic stress. It occurs in xeric sites that are more chronically moisture stressed, and at those sites that experience the most extreme stressful conditions at the time of the drought. Under this hypothesis, more intense dieback (i.e., larger and more clustered dieback patches) would be expected in areas with higher temperatures and greater evaporative demand, either in the drought period or historically; lower precipitation and actual evapotranspiration, either in the drought period or historically; and lower topographically controlled water availability and poorer stand condition. The alternative hypothesis explains drought-induced dieback as due to biotic characteristics—such as root and hydraulic architecture or stand density—that are mismatched to extreme drought conditions. This phenomenon could occur throughout the landscape, at any position on the gradient of moisture availability. However, such a mismatch is most strongly suggested when drought impacts are observed in the *least* stressful parts of the landscape, i.e., the sites with greater meteorological or topographic water availability, supporting greater tree cover and taller trees.

Although the above discussion focused on the spatial occurrence of drought impacts, we suggest that the spatial properties of drought impact patches, especially patch size and patch clustering, provide measures of drought impact intensity that are more meaningful than the presence/absence of drought impacts alone. Thus, associations between patch characteristics and environmental gradients may inform our understanding of the mechanisms underlying drought-induced dieback. Multiple regression models identified several interesting relationships between patch characteristics and environmental variables associated with moisture gradients. Although model performance was only moderate, we note that the explanatory power was substantially greater than that achieved by models of the spatial distribution of drought impact patch occurrence in this system, although slightly different environmental variables were used (R^2^ = 0.15 [82]).

In general, our results support the pattern of greater drought impacts in more xeric areas. This is evident in the modest tendency for patches to be larger at sites with lower topographic wetness index (Fig 3), and the tendency for patches to occur in denser clusters in areas of low actual evapotranspiration, short stand heights, and high summertime minimum temperatures (Fig 5). This agrees with previous research in this system that concluded that drought impact patches tended to be associated with shallow soils with limited water storage potential [82]. Under extreme drought conditions, soil water availability at these sites is expected to be very low, especially given the general long-term pattern of drying, water table lowering, and declining streamflow [55–57]. The ensuing dieback is believed to have been caused by hydraulic failure (see [83] for a review of the hypothesized mechanisms leading to drought mortality in plants). However, data on soil depths and groundwater storage potentials in the NJF is sparse (Brouwers et al. [82] estimated soil depth with a coarse proxy) and further research is needed.

The present study also identified several unexpected environmental associations of drought impact patch characteristics when expressing the meteorological conditions during the drought relative to historic averages. In particular, patch size tended to be greater when temperatures in the drought were *less extreme* relative to historic conditions. Likewise, patches were most densely clustered when drought-year precipitation was *less extreme* relative to the long-term average. The reasons behind these two patterns are unknown.

The association of the intensity of drought impacts with particular physical environments has implications for gap dynamics and landscape structure. Xeric sites most susceptible to drought impacts may be ‘chronic disturbance patches’, characterized by frequent, recurrent gap formation and recovery. Indeed, observations of stand structure suggest that the drought impact patches in the NJF have experienced previous canopy dieback events, likely caused by drought [25]. Similarly, other studies have shown that exposure to a previous drought stress makes trees more vulnerable to subsequent drought disturbances [23, 26, 84]. Ultimately, it seems likely that those sites that are chronically impacted by droughts will be the ones at which persistent land cover change to an alternative non-forested state will occur first.

Climatic disturbances, in general, may be drivers of chronic disturbance patches. For example, canopy dieback due to frost disturbance also shows specific topographic associations [52], although frost events tend to occur in opposite parts of the landscape as drought impacts in the NJF (i.e., valley bottoms vs. upslope positions). As well, microclimate conditions at gap edges may result in elevated disturbance rates, leading to persistent canopy gaps, gap expansion, or gradual ‘migration’ of gaps across a landscape [85–89]. Elevated vulnerability of edge trees, due to the harsher microclimates of forest edges relative to forest interiors, may also be a factor influencing drought impacts. In the NJF, a large proportion of drought impact patches were associated with pre-existing canopy openings [25]. In contrast, other forms of disturbance tend not to recur preferentially at the same locations. Instead, disturbances like fire or pest outbreaks require forests to recover to vulnerable stand structures before a repeated disturbance is likely to occur [90–92].

## Conclusions

Droughts, drought-induced dieback events, and their impacts to organisms, ecological communities, and ecosystems have recently gained considerable research attention. In addition, there are concerns that the frequency, duration, and severity of droughts and drought impacts may increase under global climate change. However, the impacts of drought disturbances to landscape structure and spatial ecological processes remain unknown, despite clear and consistent observations that drought-induced forest dieback often occurs in characteristically patchy distributions. We revealed that drought is indeed an important driver of landscape structure and that the spatial characteristics of drought impact patches provide useful indicators of the intensity of drought disturbance that are related to underlying environmental gradients associated with water availability.

The impacts of drought disturbance to spatial pattern have a number of implications to natural resource management. Perhaps most obviously, there is clear relevance to the widespread goal that forest management activities mimic the forest structure typical of natural disturbance regimes. In order for forest harvest practices to reflect the complex disturbance processes operating in many forests, a greater understanding of drought’s role in shaping the spatial and age structure of forests is required. In addition, knowledge of the environmental associations of drought impacts provides valuable information to the spatial prioritization of forest management. The xeric parts of the landscape currently experiencing the most extreme drought impacts (i.e., larger, more densely clustered patches of canopy dieback) are likely to be the sites where ecological regime shifts to a non-forested state are most imminent. A predictive understanding of future drought susceptibility–where it occurs on the landscape and what environmental factors it is associated with–can allow managers to proactively manage the sites most vulnerable to forest loss. By evaluating the additional ecological and cultural values of forests at vulnerable sites, managers can determine if it is acceptable to lose forest or if management effort should be invested into maintaining the presence of forest.

Future research is needed to extend the present study’s focus on spatial pattern to more explicit investigations into landscape processes. In particular, the hypothesis that drought and other climatic disturbances produce chronic disturbance patches is suggestive and should be tested. As well, further study is required to ascertain whether the spatial and temporal aspects of drought impacts are unique relative to other types of disturbance and how these spatiotemporal properties influence other ecological patterns and spatial processes, including impacts to biodiversity and other disturbance processes such as fire. Thus, we strongly encourage researchers to expand their consideration of drought impacts to the landscape scale. Impacts from drought on landscape structure and patch dynamics are likely to be considerable, yielding greater cumulative ecological impacts, via effects of landscape structure on spatial ecological processes, than are apparent from site-scale investigations.

## Supporting Information

S1 Dataset Comma-delimited spreadsheet of the centroid coordinates, area (m^2^), and perimeter (m) of delineated drought impact patches. (CSV)
